# Supplementary material for: Cellular and Molecular Network Characteristics of TARM1-Related Genes in Mycobacterium tuberculosis Infections
Source: Int J Mol Sci. 2024 Sep 20;25(18):10100. doi: 10.3390/ijms251810100 (PMC11432409; doi:10.3390/ijms251810100)
Supplement: Supplementary file 1 [file ijms-25-10100-s001.zip › Table S2. Results of GO and KEGG Enrichment Analysis for TRDEGs..pdf]

Table S2. Results of GO and KEGG Enrichment Analysis for TRDEGs.

Ontology	ID	Description	GeneRatio	BgRatio	pvalue	p.adjust
BP	GO:0019221	cytokine-mediated signaling pathway	6/16	486/18800	1.85927E-06	0.000215675
BP	GO:0071222	cellular response to lipopolysaccharide	5/16	217/18800	7.70556E-07	0.000194309
BP	GO:0071219	cellular response to molecule of bacterial origin	5/16	229/18800	1.00505E-06	0.000194309
BP	GO:0071216	cellular response to biotic stimulus	5/16	256/18800	1.73969E-06	0.000215675
BP	GO:0019221	cytokine-mediated signaling pathway	6/16	486/18800	1.86e-06	0.0002
CC	GO:0009897	external side of plasma membrane	3/16	455/19594	0.005561441	0.042722855
CC	GO:0042581	specific granule	2/16	160/19594	0.007376226	0.042722855
CC	GO:0070820	tertiary granule	2/16	164/19594	0.00773613	0.042722855
CC	GO:0097197	tetraspanin-enriched microdomain	1/16	10/19594	0.008137687	0.042722855
CC	GO:0044194	cytolytic granule	1/16	13/19594	0.0106	0.0444
MF	GO:0005125	cytokine activity	6/17	235/18410	4.46581E-08	2.50085E-06
MF	GO:0005126	cytokine receptor binding	6/17	272/18410	1.06273E-07	2.97565E-06
MF	GO:0048018	receptor ligand activity	6/17	489/18410	3.28581E-06	4.99356E-05

Ontology	ID	Description	GeneRatio	BgRatio	pvalue	p.adjust
MF	GO:0030546	signaling receptor activator activity	6/17	496/18410	3.56683E-06	4.99356E-05
MF	GO:0008009	chemokine activity	3/17	49/18410	1.17e-05	0.0001
KEGG	hsa04060	Cytokine-cytokine receptor interaction	7/11	295/8164	2.18493E-08	1.24541E-06
KEGG	hsa04061	Viral protein interaction with cytokine and cytokine receptor	5/11	100/8164	1.08684E-07	3.09751E-06
KEGG	hsa04630	JAK-STAT signaling pathway	3/11	162/8164	0.00112568	0.02138792
KEGG	hsa04062	Chemokine signaling pathway	3/11	192/8164	0.001838147	0.026193599
KEGG	hsa05133	Pertussis	2/11	76/8164	0.004455044	0.042499978

GO：Gene Ontology。BP：Biological Process。CC：Cellular Component。MF：Molecular Function。KEGG：Kyoto Encyclopedia of Genes and Genomes。TRDEGs：TARM1-Related Differentially Expressed Genes。
