# Supplementary material for: Cellular and Molecular Network Characteristics of TARM1-Related Genes in Mycobacterium tuberculosis Infections
Source: Int J Mol Sci. 2024 Sep 20;25(18):10100. doi: 10.3390/ijms251810100 (PMC11432409; doi:10.3390/ijms251810100)
Supplement: Supplementary file 1 [file ijms-25-10100-s001.zip › Table S3. Results of GSEA for Combined Datasets..pdf]

Table S3. Results of GSEA for Combined Datasets.

ID	setSize	enrichmentScore	NES	pvalue	p.adjust	qvalue
BIOCARTA_NFKB_PATHWAY	21	0.741593471	2.122681297	7.47921E-05	0.001983581	0.00174124
REACTOME_PI3K_AKT_SIGNALING_IN_CANCER	104	0.393515242	1.5494238	0.00757608	0.075747049	0.066492754
KEGG_JAK_STAT_SIGNALING_PATHWAY	146	0.575279325	2.395064543	1E-10	1.46647E-08	1.28731E-08
KEGG_HEDGEHOG_SIGNALING_PATHWAY	55	0.48282067	1.694813022	0.006871299	0.070494443	0.061881878
KEGG_MAPK_SIGNALING_PATHWAY	261	0.296582437	1.314599686	0.021763718	0.159111289	0.13967208
WP_SARSCOV2_INNATE_IMMUNITY_EVASION_AND_CELLSPECIFIC_IMMUNE_RESPONSE	65	0.7388976	2.686355	1e-10	1.47e-08	1.29e-08
WP_PROSTAGLANDIN_SIGNALING	31	0.8390993	2.653472	1e-10	1.47e-08	1.29e-08
PID_IL12_2PATHWAY	62	0.7292370	2.641289	1e-10	1.47e-08	1.29e-08

ID	setSize	enrichmentScore	NES	pvalue	p.adjust	qvalue
KEGG_CHEMOKINE_SIGNALING_PATHWAY	183	0.5903347	2.542567	1e-10	1.47e-08	1.29e-08
WP_NETWORK_MAP_OF_SARSCOV2_SIGNALING_PATHWAY	216	0.5728311	2.486833	1e-10	1.47e-08	1.29e-08

GSEA：Gene Set Enrichment Analysis。
