# Supplementary figures and images for: Cellular and Molecular Network Characteristics of TARM1-Related Genes in Mycobacterium tuberculosis Infections
Source: Int J Mol Sci. 2024 Sep 20;25(18):10100. doi: 10.3390/ijms251810100 (PMC11432409; doi:10.3390/ijms251810100)

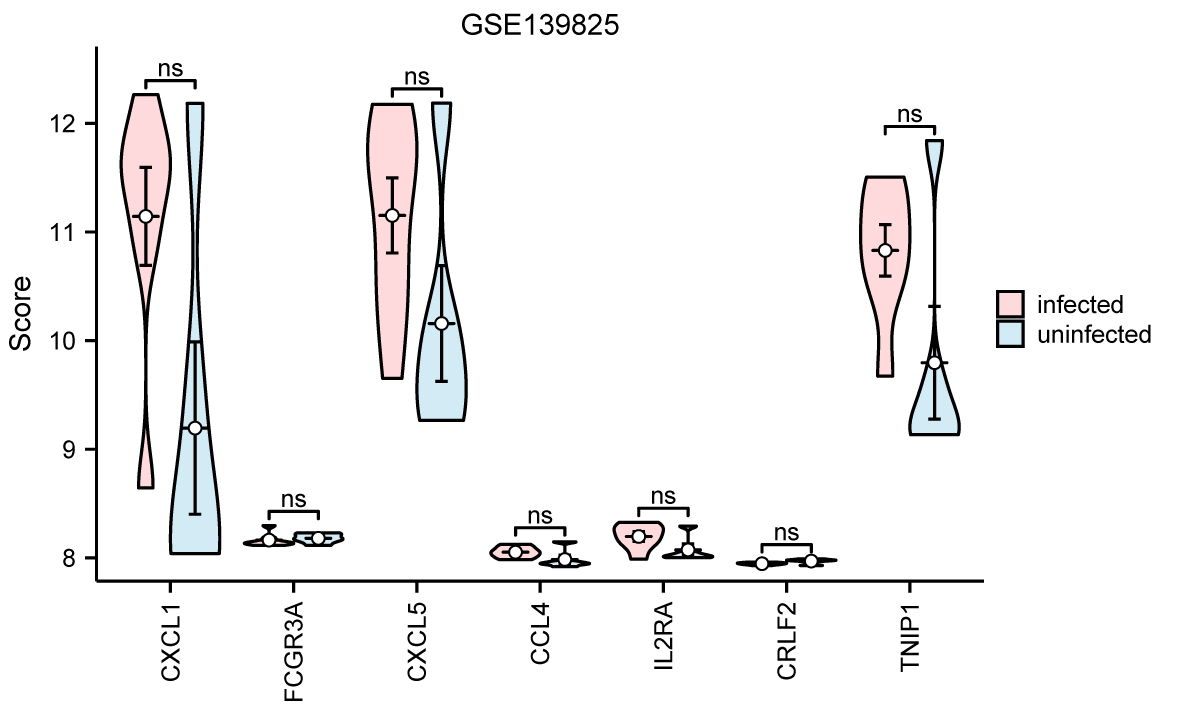

Supplement: Supplementary file 1 [file ijms-25-10100-s001.zip › Figure S1. The expression differences analysis for key genes in the GSE139825 dataset between the infected and uninfected groups. ns denotes pú╛0.05, indicating a lack of statistical significance..tif]
